# P-845. Preset Antibiotic Durations for Common Infections in Urgent Care Clinics: A Potent Antimicrobial Stewardship Tool

**DOI:** 10.1093/ofid/ofaf695.1053

**Published:** 2026-01-11

**Authors:** Elizabeth Nothdurft, Robert Paino, Nirmol Philip

**Affiliations:** St. Luke's Hospital, Chesterfield, MO; St. Luke's Hospital, Chesterfield, MO; St. Luke's Hospital, Chesterfield, MO

## Abstract

**Background:**

Unnecessary and prolonged antibiotic therapy is associated with adverse events, antibiotic resistance and cost. This study evaluated the impact of pre-set durations in the electronic health record (EHR) when ordering antimicrobials for commonly occurring infections in the Urgent Care Clinic (UCC) setting.

**Methods:**

In April 2023, we introduced pre-set durations for antibiotic prescriptions in our EHR for the most commonly occurring infections. Prescribers were able to modify pre-set durations. In addition, we sent out educational packets on the harms associated with prolonged antibiotic treatment.

We retrospectively evaluated prescribing practices for cellulitis, acute bronchitis, acute upper respiratory tract infection, sinusitis, community-acquired pneumonia, and urinary tract infection encounters among adult patients from May – December, 2022 and from May – December, 2023. Patients with prescriptions for azithromycin were excluded from the analysis. Encounters, patient characteristics, and prescription information were electronically extracted from the EHR.

For the primary outcome, we evaluated the proportion of prescriptions consistent with guideline-recommended durations. Secondary outcomes included the proportion of prescriptions with duration of 10 or more days for the above stated indications, mean antibiotic durations and change in the mean antibiotic duration for each studied indication by UCC.

**Results:**

For the indications studied, there were 2902 prescriptions for antibiotics before the intervention and 2919 prescriptions after the intervention with well-balanced baseline characteristics between the 2 groups (Table 1). Overall, the proportion of prescriptions with guideline-recommended durations increased from 34% to 64%, p < 0.001 (Figure 1). A 31% decrease in the proportion of prescriptions with a 10-day or longer duration (49% vs. 18%, p < 0.001) was attributable to the intervention (Figure 2). Antibiotic durations for all studied indications had statistically significantly decreased across all UCCs by an average of 1.39 days.

**Conclusion:**

We demonstrated that a simple, low-cost intervention in the EHR nearly doubled the number of prescriptions that aligned with national guidelines in 6 of the most commonly presenting infections in UCCs.

**Disclosures:**

Elizabeth Nothdurft, PharmD, BCPS, BCIDP, Abbvie: Honoraria Nirmol Philip, MD, MPH, Nestle: Honoraria

